# Diets Differently Regulate Tumorigenesis in Young E0771 Syngeneic Breast Cancer Mouse Model

**DOI:** 10.3390/jcm12020413

**Published:** 2023-01-04

**Authors:** Hariprasad Thangavel, Kezia Lizardo, Dhanya Dhanyalayam, Sonia De Assis, Jyothi F. Nagajyothi

**Affiliations:** 1 Center for Discovery and Innovation, Hackensack Meridian Health, Nutley, NJ 07110, USA; 2 Lombardi Comprehensive Cancer Center, Georgetown University Medical Center, Washington, DC 20057, USA

**Keywords:** diet, E0771, breast cancer, mouse, tumorigenesis, metastasis, tumor microenvironment, autophagy, apoptosis, DNA damage

## Abstract

Breast cancer (BC) is the most diagnosed cancer type, accounting for one in eight cancer diagnoses worldwide. Epidemiological studies have shown that obesity is associated with increased risk of BC in post-menopausal women, whereas adiposity reduces the risk of BC in premenopausal women. The mechanistic link between obesity and BC has been examined by combining murine BC models with high-fat diet (HFD) induced obesity. However, the effect of adiposity (not obesity) induced by a short period of HFD consumption on BC pathogenesis is not well understood. In the current study, we examined the effects of different diet compositions on BC pathogenesis using a young E0771 syngeneic BC mouse model fed on either an HFD or regular diet (RD: a low-fat high-carbohydrate diet) for a short period (4 weeks) before implanting mammary tumors in mice. We analyzed the effect of diet composition on the onset of tumor growth, metastasis, and metabolic and immune status in the tumor microenvironment (TME) using various methods including in vivo bioluminescence imaging and immunoblotting analyses. We showed for the first time that a short-term HFD delays the onset of tumorigenesis by altering the immune and metabolic signaling and energy mechanism in the TME. However, RD may increase the risk of tumorigenesis and metastasis by increasing pro-inflammatory factors in the TME in young mice. Our data suggest that diet composition, adipogenesis, and loss of body fat likely regulate the pathogenesis of BC in a manner that differs between young and post-menopausal subjects.

## 1. Introduction

Breast cancer (BC), which accounts for 12.5% of all new cancer cases annually [1], has become not only the most common cancer in women worldwide, but also the second leading cause of cancer death in women [2]. Most BC occurs in post-menopausal women and is associated with obesity [3,4]. Approximately 7% of BC occurs in women under 40 years of age while those under 30 accounts for ~1% of BC [5]. This early-onset BC is more advanced when it is diagnosed, more aggressive, and harder to treat than BC diagnosed at an older age. The risk of BC in women is strongly correlated with higher breast density and reduced breast fat for all tumor subtypes, with a stronger correlation among premenopausal women for hormone receptors-positive (ER+/PR+), human epidermal growth factor receptor 2-negative (HER2-) and triple-negative BC [6]. Although some BC is caused by inherited mutations, 85–90% of BCs are sporadic and due to acquired somatic mutations occurring in breast tissue [7]. Such somatic mutations (and epi-mutations) are thought to be caused by environmental factors, including nutritional stresses.

Epidemiologic data demonstrated that obesity is associated with an increased risk of post-menopausal BC but a decreased risk of premenopausal BC [3,8,9]. Pooled individual-level data from 758,592 premenopausal women showed that increased adiposity, particularly during early adulthood, may be associated with reductions in the risk of premenopausal breast cancer [9], suggesting that body fat mass may play different roles in regulating the risk of BC in pre- versus post-menopausal women. Many studies have been focused on assessing the effect of a high-fat diet (HFD) on the pathogenesis of BC during post-menopausal stages using various pre-clinical models and clinical studies [10,11,12,13,14,15]. Diet composition may play a major role in determining body fat mass and regulating metabolic or nutritional stress. The majority of the previous studies were mainly focused on studying the effect of obesity-induced inflammatory signaling and changes in the estrogen levels on the pathogenesis of BC using mature adult mice (>12 weeks old) treated with HFD for prolonged periods (12–16 weeks) [16,17,18,19]. However, the change in diet composition even for a short time (4–6 weeks) in growing adolescent mice may have an impact on mammary fat pad composition, development of mammary glands, and susceptibility to tumorigenesis. Therefore, in the current study, we investigated the effect of diet composition by altering the levels (kcal%) of fat and carbohydrates in the rodent diet on the pathogenesis of tumorigenesis using E0771 syngeneic BC mouse model. We used young C57BL/6J (female) mice fed on different diets for a short period of time (4 weeks) before implanting mammary tumors into mice with intact uteruses and not treated with external estrogen. Based on this study of young mammary tumor-bearing mice fed on two different (isocaloric) diets (HFD: a low-carb high-fat diet; and RD: a low-fat high-carb diet), we report for the first time that: (i) the onset of tumorigenesis and metastases was earlier in RD-fed mice and correlated with increased levels of inflammatory markers; and (ii) tumor volumes were greater in HFD-fed mice 4 weeks post tumor implantation (wpi) and the levels of DNA damage and apoptotic markers in the tumor microenvironment (TME) were increased early after tumor implantation. Our results suggest that diet plays a major role in BC pathogenesis and that a short-term HFD has a different effect in young mice compared to mature adults.

## 2. Materials and Methods

### 2.1. Cell Line and Maintenance

E0771 mouse mammary adenocarcinoma cells were originally purchased from American Type Culture Collection (ATCC) and maintained as a monolayer culture in Dulbecco’s Modification of Eagle’s Medium (#10-013-CV, Corning) supplemented with 10% heat-inactivated fetal bovine serum (#16140-071, Gibco), 1% penicillin streptomycin (#15140-122, Gibco), and 20 mM HEPES buffer solution (#15630-080, Gibco) at 37 °C in a humidified atmosphere containing 5% CO_2_. For in vivo bioluminescence imaging, E0771-tdTom/Luc cells were generated by stably transducing with tdTomato and luciferase expressing lentivirus and maintained in culture condition similar to E0771 cells [20,21].

### 2.2. In Vivo Animal Model and Research Diet

Four-week-old female C57BL/6J mice were purchased from Jackson Laboratories, Bar Harbor, ME, and housed at CDI animal research facility in sterilized filter top cages under 12-h light-dark cycle and humidity and temperature-controlled conditions. Upon arrival, the mice were randomized into two groups (n = 32/group: 10 non-tumor control mice, 16 for tumor implantation, and 6 for in vivo imaging experiment) and fed on either (i) carbohydrate-rich diet (RD: 10 kcal% fat, 70% carbohydrate, and 20% protein) (#D12450J, Research Diets, Inc., New Brunswick, NJ, USA) or (ii) fat-rich diet (HFD: 60 kcal% fat, 20% carbohydrate, and 20% protein) (#D12492, Research Diets, Inc., New Brunswick, NJ, USA) for 4 weeks prior to tumor implantation [22]. A flowchart illustrating the experimental design is presented in Supplementary Materials (Supplementary Figure S1a). All animals had ad libitum access to water and rodent research diet. Animals were routinely monitored, and the body weight was recorded twice a week for the entire duration of the study. All animal experiments were conducted in accordance with the National Institutes of Health Guide for the Care and Use of Laboratory Animals with approval from the Institutional Animal Care and Use Committee (protocol #283) of Center for Discovery and Innovation (CDI)—Hackensack University Medical Center.

### 2.3. Tumor Implantation and Measurement

Cultured cells (E0771 or E0771-tdTom-Luc) were trypsinized and harvested for tumor implantation into mice. The cells were washed with Hank’s balanced salt solution (HBSS: #14175-095, Gibco), counted, diluted at a concentration of 2.5 × 10^5^ cells/100 µL/mouse, and injected subcutaneously on the right fat pad of the fourth mammary gland using a sterile 25-gauge needle [23,24]. Tumor sizes were measured twice a week by obtaining two perpendicular dimensions using a digital caliper, where length (longest dimension) and width (dimension perpendicular to the length) were recorded until the end of study. The volume of the tumor was calculated using the following equation [25]:

(1) $$\mathrm{Tumor}\;\mathrm{volume}\;\left({\mathrm{mm}}^{3}\right)=\frac{\mathrm{length}\times {\mathrm{width}}^{2}}{2}.$$

At 5 weeks post tumor implantation, overnight fasted animals were transported to the procedure room for terminal blood collection by cardiac puncture under anesthesia. Isoflurane overdose (inhalation) was used as the preferred method of euthanasia followed by cervical dislocation. Tissue samples such as tumor, mammary gland, lung, and liver were harvested for histological and biochemical analyses. Control mice were euthanized at age 8 weeks (n = 4, pre-tumor implantation control) and at age 13 weeks (n = 6, non-tumor controls for 5 wpi tumor mice) and tissues including mammary gland, lung, and liver were harvested along with terminal blood collection. Fasting glucose was measured as described earlier [26].

### 2.4. In Vivo Bioluminescence Imaging

Bioluminescence imaging (BLI) signal intensity of E0771-tdTom-Luc tumor-bearing mice (n = 6/group; RD and HFD) was determined twice a week after tumor implantation to check for early incidence of metastasis using In Vivo Imaging Systems (IVIS: PerkinElmer, Waltham, MA, USA). Mice were administered intra-peritoneally with 3 mg of D-luciferin/mouse (#122799, PerkinElmer) and imaged after 10–15 min. BLI signal was quantified in regions of interest drawn around tumors and the whole body and the signal was expressed as photons/second [27].

### 2.5. Histological Analyses

Freshly harvested mammary fat pads and tumor tissues were fixed with 10% neutral-buffered formalin for a minimum of 48 h and then embedded in paraffin wax and sectioned for histological analyses. Hematoxylin and eosin (H & E) staining was performed on sections of healthy mammary fat pads and the images were captured as previously published [28]. Immunohistochemistry (IHC) was performed on tumor sections using rabbit polyclonal LC3A/LC3B antibody (#PA1-16931, Invitrogen, Waltham, MA, USA) and rabbit polyclonal Annexin V antibody (#8555, Cell Signaling Technology, Danvers, MA, USA) with a dilution of 1:200 and 1:300, respectively, followed by biotinylated secondary antibody using VECTASTAIN Elite ABC-HRP kit (#PK-6101, Vector Laboratories, Burlingame, CA, USA). The sections were then washed and incubated with peroxidase substrate and counterstained with hematoxylin.

### 2.6. Immunoblot Analysis

Tissue lysates from tumor and control mammary fat pads were prepared by homogenizing the tissue using a handheld homogenizer after the addition of cell lysis buffer (#9803, Cell Signaling Technology) containing Pierce protease inhibitor cocktail (#A32963, ThermoFisher Scientific, Waltham, MA, USA). The homogenate was then incubated on ice for 10 min before clarification by centrifugation for 15 min at 14,000× *g* in a cold microfuge [29,30,31]. The supernatant was recovered, and the protein concentration was quantified using Pierce BCA protein assay kit (#23225, ThermoFisher Scientific). We loaded 30 µg total protein from each sample and resolved on SDS-PAGE, and then transferred the proteins onto nitrocellulose membrane for immunoblot analysis. Primary antibodies against adiponectin (#ab22554, Abcam, Cambridge, UK), PPARγ (#2435, Cell Signaling Technology), hexokinase II (#2867, Cell Signaling Technology), phospho-AMPKα (#2535, Cell Signaling Technology), phospho-mTOR (#2971, Cell Signaling Technology), phospho-Perilipin 1 (#4856, Vala Sciences, San Diego, CA, USA), CD4 (#NBP1-19371, Novus biologicals, Englewood, CO, USA), CD8 (#NBP2-29475, Novus biologicals), F4/80 (#NB 600-404, Novus Biologicals), TNFα (#ab6671, Abcam), IL-6 (#66146-1-lg, Proteintech, Rosemont, IL, USA), IFNγ (#MM701, Invitrogen), cytochrome c (#4280, Cell Signaling Technology), SOD1 (#4266, Cell Signaling Technology), glutathione peroxidase 4 (#ab185689, Abcam), phospho-ULK1 (#6888, Cell Signaling Technology), phospho-ATR (#2853, Cell Signaling Technology), and phospho-Chk1 (#2348, Cell Signaling Technology) were used to detect the expression of corresponding proteins. Horseradish peroxidase (HRP)-conjugated anti-mouse immunoglobulin (#7076, Cell Signaling Technology), HRP-conjugated anti-rabbit immunoglobulin (#7074, Cell Signaling Technology), and HRP-conjugated anti-rat immunoglobulin (#112-035-003, Jackson ImmunoResearch, West Grove, PA, USA) were used as appropriate secondary antibodies to detect chemiluminescent signal using Invitrogen iBright Imaging Systems. β-Actin (#4967, Cell Signaling Technology) was used as control to assess equal protein loading.

### 2.7. Statistical Analysis

Data represent means ± S.E. Data were pooled, statistical analysis was performed on GraphPad Prism software version 9.4.1 using two-way ANOVA and multiple t-test as appropriate, and significant differences were determined as *p* values between <0.0001 and <0.05 as appropriate.

## 3. Results

### 3.1. Body Fat Composition and Diet Composition Alter Metabolic and Immune Status in Mammary Fat Pads in Young Mice Fed on Different Diets for a Short Period

**Figure 1:**
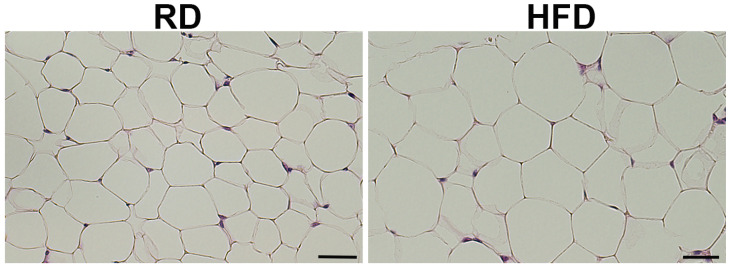
H&E-stained sections of an 8 weeks old healthy female C57BL/6J mouse mammary fat pad (n = 4/group). HFD-fed mice showed relatively bigger adipocytes in the mammary fat pad than RD mice (20× magnification, scale bar—50 µm).

To test whether diets can alter metabolic and immune status in the fat pads surrounding mammary glands, we fed C57BL/6J mice (female, 4 weeks old, n = 4/group) with two different isocaloric diets: either carbohydrate-rich RD or fat-rich HFD for a short period of 4 weeks and harvested mammary glands (8 weeks old). We measured body weight and fasting glucose levels in mice before tumor-cell implantation and found no significant changes in the levels of glucose between RD- and HFD-fed mice although the body weights of HFD-fed mice significantly increased compared to RD-fed mice (Supplementary Figure S1b). Histological sections showed slightly bigger adipocytes in the mammary fat pads of HFD-fed mice compared to RD-fed mice (Figure 1).

**Figure 2:**
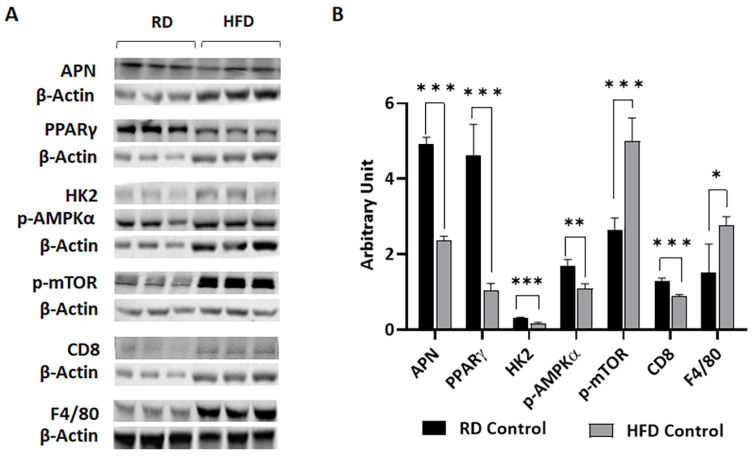
Short-term HFD consumption decreased the expression levels of adiponectin (APN), PPARγ, hexokinase II (HK2), p-AMPKα, and CD8 and increased the levels of p-mTOR and F4/80 in the mammary fat pads of healthy female C57BL/6J mice compared to mice fed on a carbohydrate-rich RD. (**A**) Immunoblots showing different protein expression between RD- and HFD-fed healthy mice. β-Actin was used as loading control. (**B**) Bar graphs showing the levels of each protein marker normalized to β-Actin. The error bars represent the standard error of the mean. * *p* ≤ 0.05, ** *p* < 0.005, and *** *p* ≤ 0.0001.

We analyzed the markers of adipogenesis (adiponectin and PPARγ), glycolysis (hexokinase II), energy signaling (p-AMPKα), cell proliferation (p-mTOR), and immune cells (CD8, macrophage F4/80) in mammary fat pad by immunoblotting (Figure 2). Except for the levels of macrophages (F4/80) and cell proliferation marker (p-mTOR), all the other markers analyzed were significantly decreased in HFD-fed mice compared to RD-fed mice (Figure 2). Decreased p-AMPKα and increased p-mTOR in HFD mice suggest that the cells were not energy depleted and were proliferating in the mammary fat pad. These data showed that carbohydrate- and fat-rich diets regulate the metabolic and immune status of the mammary fat pad.

### 3.2. HFD and RD Differently Regulate Tumorigenesis in E0771 Syngeneic BC Mouse Model

**Figure 3:**
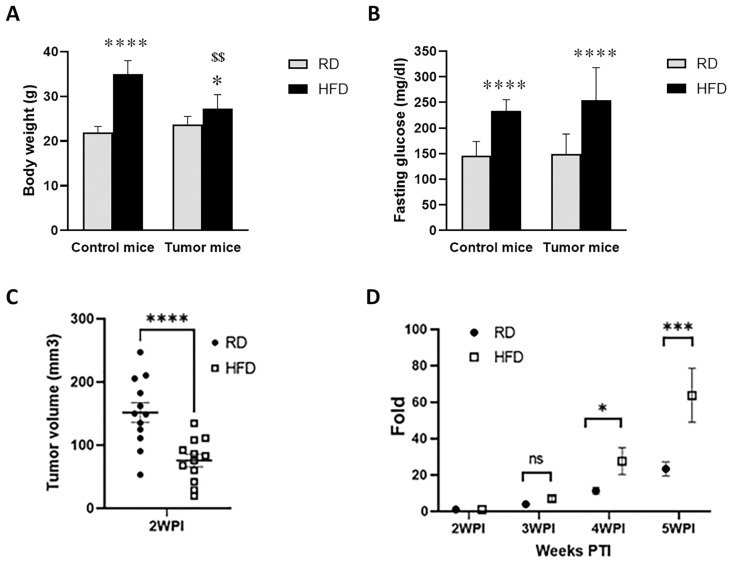
Effect of RD and HFD on body weight, fasting plasma glucose, and tumorigenesis. Bar graphs showing body weights (**A**) and fasting plasma glucose levels (**B**) of RD- and HFD-fed tumor bearing mice (5 wpi) and age-matched control mice at 5 wpi. The error bars represent the standard error of the mean. * *p* ≤ 0.05 and **** *p* ≤ 0.0001 between RD and HFD groups. ^$$^ *p* ≤ 0.01 with respect to control mice. Short-term feeding on RD and HFD differently regulates the early onset of (**C**) tumorigenesis and (**D**) tumor progression in young E0771 syngeneic BC mice (n = 16/group). C57BL/6J (female, 4 weeks old) mice fed a RD or HFD for 4 weeks prior to tumor implantation were continued on the appropriate diet. (**C**) RD tumors were significantly larger compared to HFD tumors at 2 wpi. (**D**) HFD showed significantly increased tumor growth after 3 wpi compared to RD. Graph shows significant fold increase in tumor volume in HFD mice compared to RD mice between 2 and 5 wpi. *Y*-axis shows fold change in tumor volume compared to tumor volume at 2 wpi. Data represents mean +/− SEM fold change in tumor volume at different wpi. ns – not significant, * *p* < 0.05, *** *p* ≤ 0.001 and **** *p* < 0.0001.

As demonstrated above, the environment (metabolic and immune) in the mammary fat pad was altered in mice by short-term HFD or RD. To test whether the altered metabolic and immune environment in the mammary fat pad will affect tumorigenesis and tumor progression in young mice, we used a well-established E0771 syngeneic BC model. Unlike other diet-induced obesity models of BC, our mice were young, fed on HFD for a short period of 4 weeks, and the levels of estrogen were not manipulated (i.e., the uterus was intact and estrogen pellets were not inserted). We subcutaneously injected 2.5 × 10^5^ E0771 cells into the right fat pad of the fourth mammary gland of female C57BL/6J mice (8-weeks old, n = 16/group), and tumor growth was measured twice weekly as described in the methods section. The body weights of non-tumor control HFD-fed mice significantly increased compared to control RD-fed mice at 13 weeks age (Figure 3A). The body weights of tumor implanted HFD-fed mice also significantly increased compared to tumor implanted RD-fed mice at 5 wpi. Interestingly, the body weights of tumor implanted HFD-fed mice significantly decreased at 5 wpi compared to the age matched control HFD-fed mice (Figure 3A). We did not see a significant difference in body weight between tumor bearing RD-fed mice and the age matched control RD-fed mice at 5 wpi (Figure 3A). We also analyzed glucose levels in mice at 5 wpi. Although the control mice fed on HFD for 4 weeks showed a significant weight gain but no significant difference in their fasting glucose levels compared to control RD-fed mice, we found a significant increase in glucose levels and body weight in control HFD-fed mice at 13 weeks old. We observed a significant increase in fasting glucose levels in tumor bearing HFD-fed mice compared to tumor bearing RD-fed mice at 5 wpi (Figure 3B). Interestingly, no significant difference was observed in fasting glucose levels between tumor bearing RD-fed mice and control RD-fed mice at 5 wpi (Figure 3B). Therefore, prior to tumor cell implantation, these HFD-fed mice were neither obese nor metabolically dysfunctional. However, the post-tumor HFD-mice were non-obese hyperglycemic at 5 wpi compared to obese-hyperglycemic control HFD-fed mice (Figure 3A,B). The mice developed tumors by 2 wpi, and the tumors were significantly larger in RD-fed mice compared to HFD-fed mice (Figure 3C). However, the tumors in HFD-fed mice were faster-growing, surpassing the tumors in RD-fed mice by 5 wpi (average size of 4163 ± 599 mm^3^ vs. 3290 ± 499 mm^3^). We found that the fold increase in tumor volume (from 2 wpi) was significant in HFD-fed mice at 4 and 5 wpi compared to RD-fed mice (Figure 3D).

**Figure 4:**
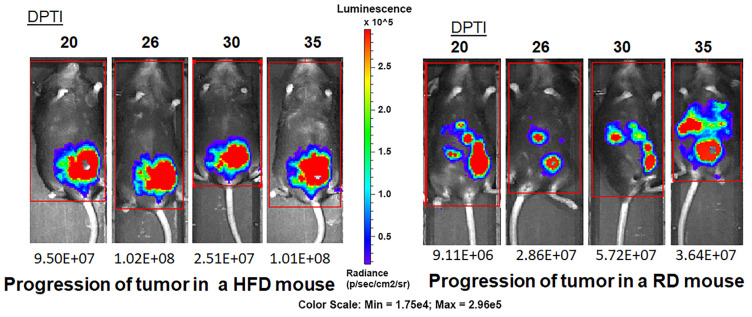
Bioluminescence imaging by IVIS showed distant metastases in RD-fed mice (**right** panel) at earlier point after tumor implantation compared to HFD mice. The primary tumors were relatively larger in size in HFD mice (**left** panel) starting as early as 20 days post tumor implantation (DPTI). C57BL/6J (female, 4 weeks old, n = 6/group) mice fed on RD or HFD for a short period of 4 weeks were subcutaneously injected with luciferase expressing E0771-tdTom-Luc (2.5 × 10^5^) cells on the right fat pad of the fourth mammary gland and imaged twice a week after tumor implantation.

E0771 cells often metastasize to distant sites, such as the intestinal mesentery, diaphragm, peritoneal wall, and the lung [23,32,33,34,35,36]. Therefore, we euthanized RD- and HFD-fed tumor mice at 5 wpi and observed a higher rate of metastasis (in the lungs, intraperitoneal region, and intestine) in RD-fed mice (65%) compared to HFD-fed mice (35%) using a dissection microscope (data not shown). To further examine whether RD increases the chance of early incidence of metastasis, we performed a pilot experiment using luciferase expressing E0771 cells and measured the rate of metastasis by implanting E0771-tdTom-Luc cells similarly in RD- and HFD-fed female C57BL/6J mice (Figure 4**)**. IVIS imaging analysis demonstrated early metastases in regions of the lungs, peritoneal cavity, and liver in RD mice as early as 20 days post tumor implantation (DPTI), whereas the HFD-fed mice showed a solid primary tumor in the mammary pad (Figure 4). After 30 DPTI, some of the HFD-fed mice also showed distant metastases (data not shown). These data suggest that RD-fed mice are more prone to develop early metastases compared to HFD-fed mice and HFD-fed mice tend to develop bigger tumors compared to RD-fed mice at the later stage of tumor implantation.

### 3.3. HFD and RD Provide Different Metabolic and Immune Enviroments for the Growing Tumor

**Figure 5:**
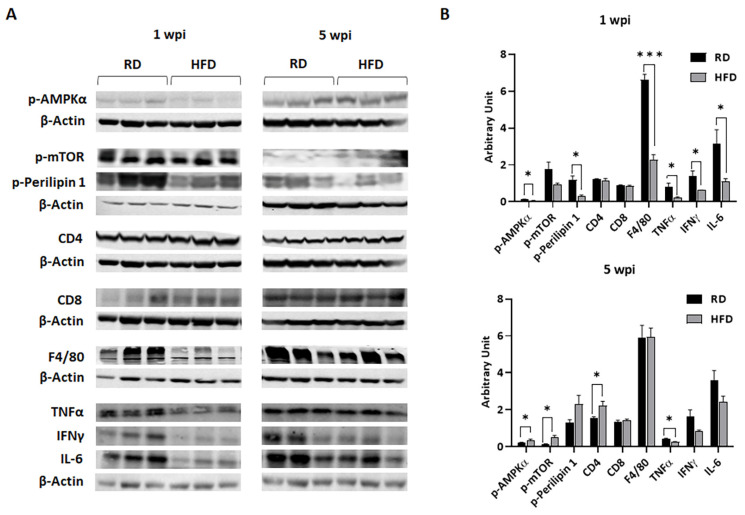
RD and HFD differently regulate the expression levels of p-AMPKα, p-mTOR, p-Perilipin 1, CD4, CD8, F4/80, TNFα, IFNγ, and IL-6 in tumor-associated mammary fat pad during early (1 wpi) and later (5 wpi) time points of tumor growth in young female E0771 tumor-bearing mice. (**A**) Immunoblots showing different protein expression between RD and HFD at 1 wpi and 5 wpi. β-Actin was used as loading control. (**B**) Bar graphs showing the levels of each protein marker normalized to β-Actin (top panel—1 wpi; bottom panel—5 wpi). The error bars represent the standard error of the mean. * *p* ≤ 0.05 and *** *p* ≤ 0.001.

Our data showed an early onset of tumorigenesis and an early incidence and rate of metastases in RD-fed mice, which was higher compared to HFD-fed young mice (Figure 3 and Figure 4). On the other hand, the fold change in tumor size at later stages of tumor growth (between 4 and 5 wpi) was significantly higher in HFD-fed mice compared to RD mice (Figure 3). We also showed that the metabolic and immune environment in the mammary fat pad differed between mice fed on RD vs. HFD before implanting tumor cells. To examine whether the pre-existing metabolic and immune environment in the mammary fat pad (due to diet) can differently regulate tumor growth by facilitating either early dissociation of tumor cells or tumor proliferation, we analyzed the metabolic and immune environment in tumor associated fat pads at early and late stages of tumor growth (1 wpi and 5 wpi, respectively). Immunoblotting analysis was performed on the lysates of tumor mammary pads to analyze the levels of metabolic and proliferation markers such as p-AMPKα and p-mTOR, a marker of lipolysis of lipid droplets (p-Perilipin 1), immune cells (CD4, CD8, and macrophages F4/80), and inflammatory markers (TNFα, IFNƳ, and IL-6) (Figure 5). The levels of p-AMPKα significantly decreased at 1 wpi and significantly increased at 5 wpi in tumor associated mammary pads in HFD-fed mice compared to RD-fed mice (Figure 5). The levels of p-mTOR were not altered in tumors between RD- and HFD-fed mice at 1 wpi but were increased in HFD mice tumors compared to RD-fed mice at 5 wpi (Figure 5). The levels of p-Perilipin 1 significantly decreased in HFD-fed mouse tumors compared to RD-fed mice at 1 wpi and were unchanged at 5 wpi (Figure 5). Interestingly, the levels of p-AMPKα significantly increased and p-mTOR significantly decreased in both RD- and HFD-fed mouse tumors at 5 wpi compared to 1 wpi (Supplementary Figure S2). The levels of p-Perilipin 1 were not changed in tumors of RD-fed mice between 1 and 5 wpi; however, they significantly increased in HFD-fed mice between 1 and 5 wpi (Supplementary Figure S2). In sum, our data showed a significant difference in the TME metabolic status between RD- and HFD-fed mice.

The levels of CD4 and CD8 T-cells were not altered in tumors between RD- and HFD-fed mice at 1 wpi; however, the levels of F4/80 (macrophages) significantly increased in RD-fed mice compared to HFD-fed mice at 1 wpi (Figure 5). The levels of infiltrated immune CD4 and CD8 cells in both RD- and HFD-fed mice tumors significantly increased at 5 wpi compared to 1 wpi (Supplementary Figure S3). Interestingly, the levels of macrophages significantly increased in HFD-fed mouse tumors at the later time point of tumorigenesis (5 wpi) compared to early tumorigenesis (1 wpi) (Supplementary Figure S3). These data demonstrated that increased infiltration of macrophages occurs at the early time point of tumorigenesis in RD-fed mice, whereas in HFD-fed mice, the infiltration of macrophages increases only at the later stages of tumorigenesis. The levels of pro-inflammatory cytokines such as TNFα, IFNƳ, and IL-6 significantly increased in tumors of RD-fed mice compared to HFD-fed mice at 1 wpi (Figure 5). However, at 5 wpi, the pro-inflammatory cytokine levels significantly increased in tumors of HFD-fed mice compared to 1 wpi (Supplementary Figure S4). Thus, we find that early infiltration of macrophages in RD mice tumors and delayed infiltration of macrophages in HFD mice tumors may cause different inflammatory TME in RD-fed mice vs. HFD-fed mice.

### 3.4. Diets Differently Regulate Energy Metabolism in Breast TME in E0771 Tumor-Bearing Mice during Early Tumorigenesis and Late Tumor Progression

**Figure 6:**
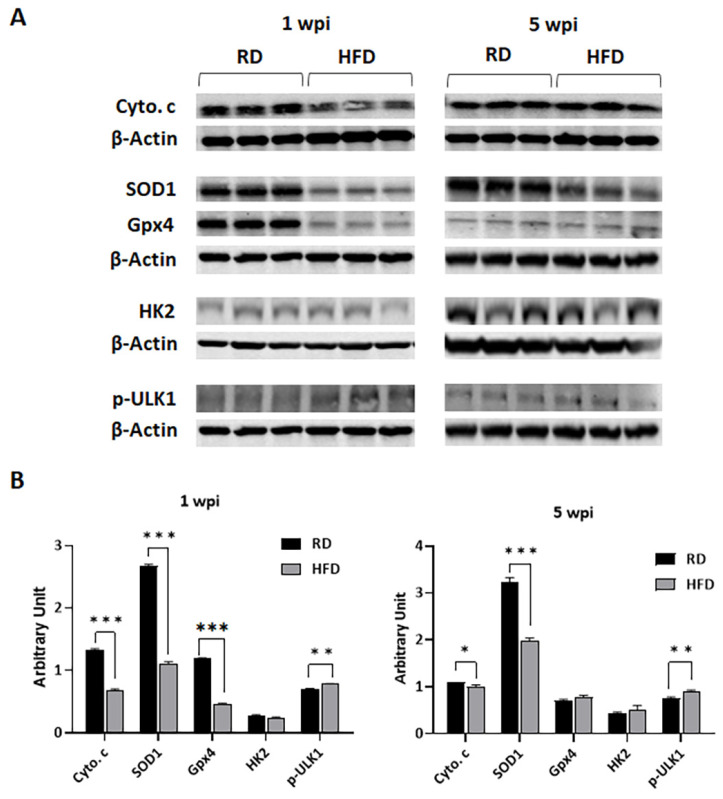
RD and HFD differently regulate the expression levels of markers of mitochondrial β-oxidation (cytochrome c and SOD1), glycolysis (HK2), and autophagy (p-ULK1) in tumor-associated mammary fat pads during early (1 wpi) and later (5 wpi) time points of tumor growth in young female E0771 tumor-bearing mice. (**A**) Immunoblots showing different protein expression levels between RD and HFD at 1 wpi and 5 wpi. β-Actin was used as loading control. (**B**) Bar graphs showing the levels of each protein marker normalized to β-Actin (left panel—1 wpi; right panel—5 wpi). The error bars represent the standard error of the mean. * *p* < 0.05, ** *p* < 0.005, and *** *p* ≤ 0.001.

Carbohydrate-rich RD and fat-rich HFD may differently alter energy metabolism in TME by regulating the levels of mitochondrial oxidative phosphorylation and glycolysis. We measured the levels of markers of mitochondrial β-oxidation, such as cytochrome c, superoxide dismutase 1 (SOD1), and glutathione peroxidase 4 (Gpx4), and a marker of glycolysis, hexokinase II (HK2), by immunoblotting analysis (Figure 6). The levels of cytochrome c and SOD1 significantly increased in tumor associated mammary pads in RD-fed mice compared to HFD-fed mice at 1 and 5 wpi (Figure 6). The levels of cytochrome c and SOD1 significantly increased in HFD-fed mice tumors at 5 wpi compared to 1 wpi (Figure 6). However, the levels of SOD1 did not increase, correlating to the levels of increased cytochrome c in HFD-fed mice (as compared to the cytochrome c and SOD1 levels in RD-fed groups), suggesting that the TME in HFD-fed mice is likely under oxidative stress at 5 wpi (Supplementary Figure S5). The levels of another oxidative stress marker Gpx4 significantly decreased in the TME in HFD-fed mice compared to RD-fed mice at 1 wpi and showed no significant difference between the groups at 5 wpi (Figure 6). The levels of HK2 were not significantly altered between RD- and HFD-fed mouse tumors either at 1 or 5 wpi. However, the levels of oxidative phosphorylation marker cytochrome C significantly decreased in RD-fed mice at 5 wpi compared to 1 wpi, whereas its levels significantly increased in HFD-fed mice at 5 wpi compared to 1 wpi (Supplementary Figure S5). Thus, our data suggested that both basal glycolysis and oxidative phosphorylation are major energy sources at an early time point after tumor implantation in RD-fed mice, and that oxidative phosphorylation is the major energy source at a later time point after tumor implantation in HFD-fed mice.

### 3.5. Autophagy and Apoptosis Increase in Tumor Associated Mammary Fat Pads in HFD-Fed Mice at Early Stages of Tumorigenesis

**Figure 7:**
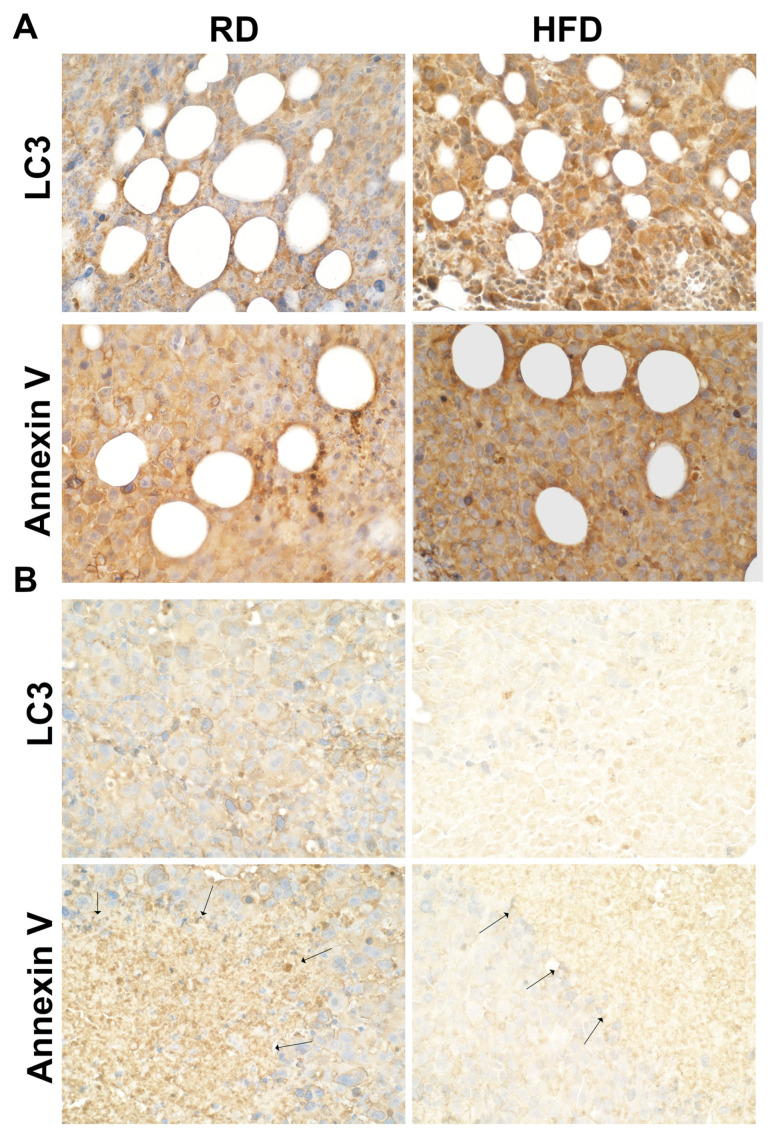
HFD increases autophagic apoptosis at the early tumor point and decreases it at a later stage of tumor growth as demonstrated by IHC in tumor sections. (**A**) The markers of autophagy (LC3) and apoptosis (Annexin V) significantly increased in short-term HFD-fed mice compared to RD-fed mice at 2 wpi; (**B**) however, the levels of these markers significantly decreased at 5 wpi in both RD- and HFD-fed mice compared to 2 wpi (black arrows indicate the invasive front associated with adipocytes) (40× magnification).

Many studies have shown that glucose is essential for the growth of tumor cells. Our data showed decreased levels of mitochondrial oxidative phosphorylation and increased levels of cellular energy sensor (p-AMPKα) in the TME in HFD-fed mice (low in carbohydrate) compared to RD-fed mice, which suggests a high energy demand in the growing TME in HFD mice. To identify whether autophagy and apoptosis are induced in the TME to satisfy the essential energy demands of the growing tumor cells, we performed immunoblotting and IHC analyses as described in the Materials and Methods section using the markers of autophagy (phosphorylated ULK1 and LC3) and apoptosis (Annexin V). Immunoblotting analysis (Figure 6) showed increased p-ULK1 in HFD-fed mice compared to RD-fed mice at both 1 wpi and 5 wpi. IHC analysis demonstrated that at an early time point of tumorigenesis (2 wpi) the levels of LC3 and Annexin V were significantly increased in HFD-fed mice tumors compared to RD-mice (Figure 7). The levels of LC3 and Annexin V were greater around the adipocytes associated with tumors and at the invasive front (Figure 7). However, the levels of LC3 and Annexin V significantly decreased in both the RD- and HFD-fed mice at 5 wpi compared to 2 wpi. We found that autophagy and apoptosis were increased not only in the tumor associated adipocytes but also in the tumor cells in HFD-fed mice at the early time points of tumorigenesis, which likely caused the decreased tumor size observed at 2 wpi in HFD-fed mice compared to RD-fed mice (Figure 3C).

### 3.6. HFD Increases DNA Damage in TME at Early Stages of Tumorigenesis

**Figure 8:**
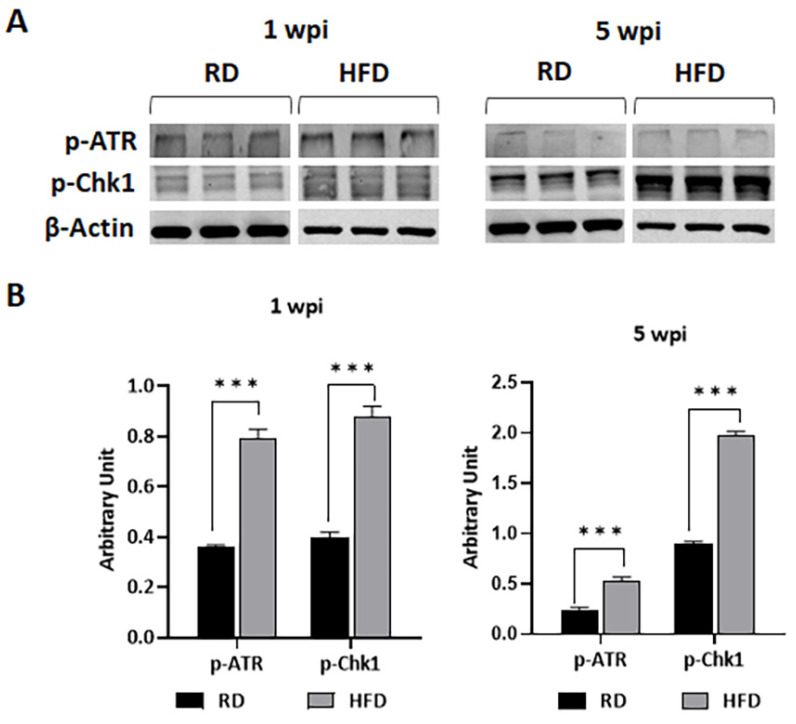
RD and HFD differently regulate the markers of DNA damage (p-ATR and p-Chk1) in tumor-associated mammary fat pads during early (1 wpi) and later (5 wpi) time points of tumor growth in young female E0771 tumor-bearing mice. (**A**) Immunoblots showing different protein expression levels between RD and HFD at 1 wpi and 5 wpi. β-Actin was used as loading control. (**B**) Bar graphs showing the levels of each protein marker normalized to β-Actin (**left** panel—1 wpi; **right** panel—5 wpi). The error bars represent the standard error of the mean. *** *p* ≤ 0.001.

Finally, we analyzed whether autophagy is activated in response to DNA damage in HFD-fed mice. We measured the levels of DNA damage markers such as p-ATR and p-Chk1 in the tumor lysates by immunoblotting analysis. DNA damage or replication stress activates ATR by phosphorylation, which in turn activates its effector kinase Chk1 [37]. The levels of both p-ATR and p-Chk1 significantly increased in HFD-fed mice tumors compared to RD mice both at 1 and 5 wpi (Figure 8). The levels of p-Chk1 further significantly increased at 5 wpi compared to 1 wpi in both RD- and HFD-fed mice (Supplementary Figure S6). Thus, we find that DNA damage sensing mechanisms are highly upregulated in tumors in HFD-fed mice compared to RD-fed mice.

## 4. Discussion

Epidemiology studies indicate that increased body fat is associated with increased risk of BC in post-menopausal women, whereas increased adiposity reduces the risk of BC in young and premenopausal women [3,8,9]. The challenge is to understand why body fat plays different roles in contributing to the risk of BC pathogenesis in pre- vs. post-menopause women. Diets mainly rich in fat increase body fat mass and alter the composition and physiology of adipose tissue, including at the breast stromal site. Many studies have explored the chronic effects of HFD-induced obesity on tumorigenesis and metastasis in the pathogenesis of BC using various mouse mammary tumor models, such as E0771 and MMTV-PyMT [17,18]. Earlier studies have demonstrated that feeding mice a HFD for 12–16 weeks induces not just obesity but also increases pro-inflammatory cytokines in the mammary fat pad [16,17], tumor lipogenesis [18], and estrogen levels [19], which are associated with high risk of tumor progression and metastasis in BC. However, few studies have explored the effect of mild adiposity on the development of tumorigenesis in young mice. In this study, we have identified the effects of a short-term HFD consumption started during puberty on BC pathogenesis and cellular signaling pathways using young E0771 tumor-bearing mice and compared the effects of the HFD with a carbohydrate-rich RD. Our data demonstrate for the first time that mild adipogenesis in the breast induced by HFD delays tumorigenesis and prevents early metastasis. We also show for the first time that RD induces an acute pro-inflammatory flux into the TME, as well as dissociation of tumor cells, causing metastasis during early tumor development stages. However, we also found that HFD stimulates tumor growth at later stages after tumor implantation, resulting in larger tumors compared to RD-fed mice. Together, these results reveal how different diet compositions can differently alter energy metabolism, inflammatory signaling, and cell death (autophagy/apoptosis) in TME and influence tumor growth/progression.

We and others previously demonstrated that under pathological conditions, high-carbohydrate RD promotes inflammation [38,39,40,41]. In addition, it has been shown that the pro-inflammatory transcription factor NF-κB plays a crucial role in the metabolic profile of pediatric sarcomas, potentially through the regulation of glycolysis (HK2) [42]. Our data (Figure 5) suggest that increased glycolysis promotes a pro-inflammatory environment and an early onset of tumor growth in RD mice compared to HFD mice. Pro-inflammatory cytokines, such as TNF-α, IFNγ and IL-6, promote tumorigenesis and metastasis [43,44,45,46]. Our data suggest that RD-induced pro-inflammatory status in the TME is likely the cause for the observed early onset of tumor development and metastasis in RD-fed mice compared to HFD-fed mice. In support of this conclusion, we found a significant increase in pro-inflammatory cytokines, such as IFNγ and IL-6, at the later stages of tumor growth compared to early stages in HFD-fed mice, correlating with the increased tumor size in HFD-fed mice at 5 wpi. Many studies have shown that obesity induced proinflammatory cytokines promote metastasis in post-menopausal breast cancer [47,48]. Using diet-induced obesity murine models of E0771 breast cancer (aged above 14 weeks old at the time of tumor inoculation), it has been shown that HFD increases the levels of proinflammatory cytokines in the TME, which correlates with increased tumor size and lung metastasis [49,50,51,52]. However, in our E0771 implanted pubertal breast cancer model, a short-term HFD feeding in fact increased the latency of tumorigenesis compared to RD, which can be attributed to increased proinflammatory cytokines in the TEM in RD-fed mice. Interestingly, in an MMTV-PyMT breast cancer model, feeding an HFD enhanced tumorigenesis and lung metastasis while also increasing the latency of tumorigenesis compared to mice fed an RD [53], which is similar to our observation in the E0771 model. Our data indicated that a slight increase in adiposity in the breast in young adults may prevent a proinflammatory environment. Because the propensity for metastasis is governed by many physiological factors and cellular mechanisms including invasion, dissemination, and survival of tumor cells outside the initial primary tumor microenvironment, further studies are warranted to evaluate how diets can regulate the cellular mechanisms involved in invasion, dissemination, and survival of tumor cells in distant sites.

Fast-growing tumor cells need an efficient ATP supply in the TME. AMPK is a crucial cellular energy sensor, and it becomes activated when cellular ATP levels decrease due to a variety of physiological stresses [54]. AMPK signaling coordinates cellular metabolism in tumor cells, including cell growth, energy metabolism (fatty acid catabolism, protein synthesis, and glucose uptake), and autophagy [54]. Many studies using the AMPK-activating drug metformin have shown that activation of AMPK can reduce tumor growth by promoting anti-inflammation [55]. Activated AMPK also inhibits mTOR-C1 signaling by activating TSC activity, thereby inhibiting the mTOR pathway, which is a promotor of tumor cell growth in breast cancer [56]. However, in our breast cancer model, we observed increased p-AMPK levels associated with increased p-mTOR and pro-inflammatory signaling in the TME in RD-fed mice and HFD-fed mice at an early time point of tumorigenesis and at a late time point of tumor progression, respectively (Figure 5). These data suggest that the activation of AMPK is essential in growing tumor cells to promote the necessary energy demands (uptake of glucose, fatty acid oxidation) and protein biosynthesis and to prevent metabolic stress (i.e., to reduce the lipotoxic effects of intracellular lipid accumulation by elevating lipid catabolism). It has been shown that metformin can promote the persistence of estrogen-deprived cells and tumors via increased mitochondrial respiration driven by fatty acid oxidation [57].

Our data demonstrate that a short-term feeding (for 4 weeks) of HFD increases body weight without causing hyperglycemia in 8-week-old mice; however, a long-term feeding (for 9 weeks) not only increases body weight but also causes hyperglycemia in control mice. Interestingly, in tumor bearing mice, a long-term feeding of a HFD causes non-obese hyperglycemia, which suggests a significant loss of body fat. It has been shown that acute adipocyte lipolysis causes loss of body fat and increases hyperglycemia [58,59]. It should be noted that hyperglycemia is a risk factor for tumor progression [60]. Given that the cancer-associated adipocytes undergo lipolysis and provide the major source of energy for the growing tumor in breast cancer [61], our data indicate that a significant loss of body weight in HFD-fed mice during the later time point of tumor implantation, might have caused hyperglycemia (non-obese hyperglycemia) and associated increase in pro-inflammatory signaling and tumor growth.

We showed that RD and HFD create different metabolic environments in the breast microenvironment (e.g., increased glycolysis (HK2) and p-AMPK in RD-fed mice compared to HFD-fed mice), which may differently influence energy metabolism in the TME during tumorigenesis and replication of tumor cells. Oxidative glycolysis is a well-accepted energy mechanism in tumor cells, which would rapidly provide ATP to the growing tumor cells [62,63]. Our data demonstrated a high level of oxidative phosphorylation in the TME at early time points after tumor implantation in RD-fed mice due to increased levels of lipid hydrolysis (phospho-perilipin) and fatty acid oxidation (p-AMPK), which likely provided more ATP in the TME and caused early onset of tumor growth. However, the levels of oxidative phosphorylation were elevated at later time points after tumor implantation, which likely caused increased tumor growth compared to the early time point in HFD-fed mice. Increased oxidative phosphorylation releases increased intracellular reactive oxygen species (ROS) [64] and causes increased oxidative stress in the absence of sufficient antioxidant factors (e.g., SOD1, Gpx4) and results in autophagy and apoptosis [65,66,67,68]. Our data indicated that the levels of antioxidants (such as SOD1, Gpx4) in the TME of HFD-fed mice might be insufficient to protect the cells from autophagy and apoptosis during the process of tumor development, at least during the early time point after tumor implantation. However, increased oxidative stress can also cause DNA damage [69], which is indicated by the increased levels of the markers of DNA damage, autophagy, and apoptosis in HFD-fed mice at later time point after tumor implantation. Many studies have shown that the starvation of nutrients including glucose in mammalian cells can induce autophagy [70]. The cellular nutritional stress can also induce autophagy [71]. Autophagy is crucial in balancing nutrient and energy supply and demand for the survival and function of cells in response to environmental stress [72]. We observed increased autophagic signaling in the TME in the early stages of tumorigenesis in HFD-fed mice (Figure 7). Although circulating glucose levels in HFD-fed mice were not significantly altered compared to RD-fed mice, the growing tumor cells in the TME must adapt to the lipid-rich environment, which could initially cause nutritional stress in HFD-fed mice. Once the tumor cells adapt to the lipid-rich nutritional environment, the growth of tumors likely rapidly increased in HFD-fed mice. Altogether, our data suggested that diet composition modulates the nutritional environment and energy metabolism, which in turn play a major role in dictating the onset of tumorigenesis and tumor progression.

## 5. Conclusions

It has been shown that being overweight and obese are associated with higher risks of post-menopausal breast cancer. Fat tissue produces excess amounts of estrogen, high levels of which have been associated with an increased risk of ER+ breast cancer in obese post-menopausal women. On the other hand, in premenopausal women, adiposity reduces the risk of breast cancer. Our study demonstrated that a slight increase in adiposity induced by feeding young mice with a HFD for a short time period (4–6 weeks) before tumor implantation delays the onset of tumorigenesis by altering the immune and metabolic signaling and energy mechanism in the TME. In contrast, carbohydrate-rich diets induced a nutritional status in the breast, which may increase the risk of tumorigenesis and metastasis by increasing the pro-inflammatory factors in the TME of young mice. Our data suggest that energy intake, diet composition, adipogenesis, and loss of body fat play important roles in the pathogenesis of BC, which may differ between young and adult subjects. In order to decrease the risk of breast cancer in young adults who are likely to be conscious of body weight, further studies are warranted to understand how nutritional stress (undernutrition and overnutrition) can impact the onset and progression of breast primary tumors.

## Data Availability

Not applicable.

## Supplementary Materials

The following supporting information can be downloaded at: https://www.mdpi.com/article/10.3390/jcm12020413/s1, Figure S1: (a) Flowchart of the experimental design. (b) Bar graphs showing the change in body weight and fasting glucose levels in control mice after 4 weeks feeding on HFD compared to RD. The error bars represent the standard error of the mean. **** *p* ≤ 0.0001 and no significance (NS) compared to RD mice. Figure S2: Bar graphs showing the levels of p-AMPKα, p-mTOR and p-Perilipin 1 normalized to β-Actin between 1 wpi and 5 wpi in both RD (left) and HFD (right) mice tumors. The error bars represent the standard error of the mean. * *p* ≤ 0.05, ** *p* < 0.005 and *** *p* ≤ 0.001. Figure S3: Bar graphs showing the levels of infiltrated immune cells (CD4, CD8 and F4/80 macrophages) normalized to β-Actin between 1 wpi and 5 wpi in both RD (left) and HFD (right) mice tumors. The error bars represent the standard error of the mean. * *p* ≤ 0.05, ** *p* < 0.005 and *** *p* ≤ 0.001. Figure S4: Bar graphs showing the levels of inflammatory markers (TNFα, IFNγ and IL-6) normalized to β-Actin between 1 wpi and 5 wpi in both RD (left) and HFD (right) mice tumors. The error bars represent the standard error of the mean. * *p* ≤ 0.05 and ** *p* ≤ 0.005. Figure S5: Bar graph showing the protein expression levels of energy metabolism (oxidative phosphorylation vs. glycolysis) normalized to β-Actin between 1 wpi and 5 wpi in both RD (left) and HFD (right) mice tumors. The error bars represent the standard error of the mean. * *p* ≤ 0.05 and ** *p* ≤ 0.005. Figure S6: Bar graph showing the protein expression levels of DNA damage marker (p-Chk1 and p-ATR) normalized to β-Actin between 1 wpi and 5 wpi in both RD and HFD mice tumors. The error bars represent the standard error of the mean. *** *p* ≤ 0.001.
