# Supplementary material for: Diets Differently Regulate Tumorigenesis in Young E0771 Syngeneic Breast Cancer Mouse Model
Source: J Clin Med. 2023 Jan 4;12(2):413. doi: 10.3390/jcm12020413 (PMC9862441; doi:10.3390/jcm12020413)
Supplement: Supplementary file 1 [file jcm-12-00413-s001.zip › jcm-2068370-supplementary.pdf]

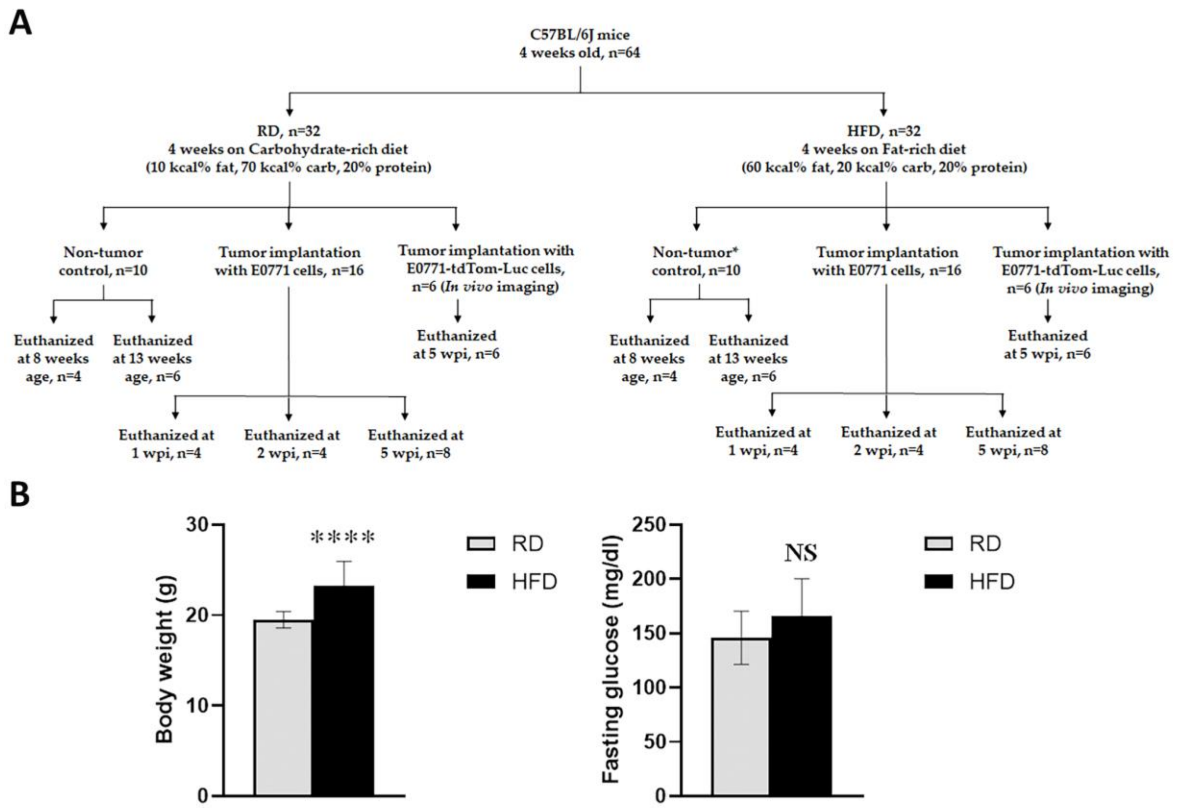

Figure S1: (a) Flowchart of the experimental design. (b) Bar graphs showing the change in body weight and fasting glucose levels in control mice after 4 weeks feeding on HFD compared to RD. The error bars represent the standard error of the mean. **** $p \leqslant 0.0001$ and no significance (NS) compared to RD mice.

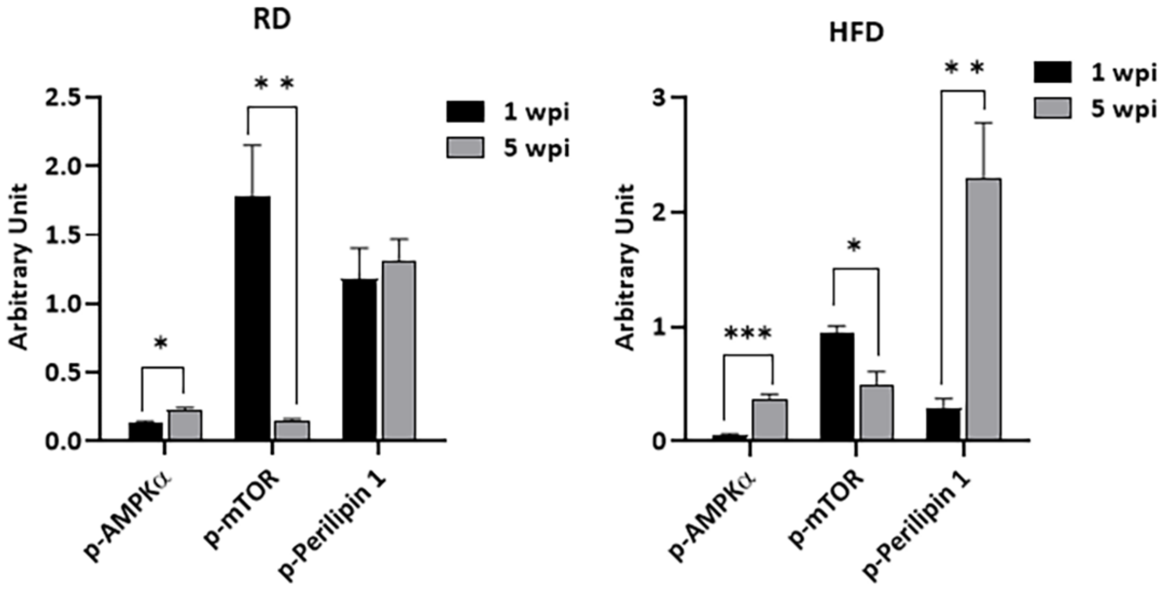

Figure S2: Bar graphs showing the levels of p-AMPKα, p-mTOR and p-Perilipin 1 normalized to β-Actin between 1 wpi and 5 wpi in both RD (left) and HFD (right) mice tumors. The error bars represent the standard error of the mean. * $p \leq 0.05$, ** $p < 0.005$ and *** $p \leq 0.001$.

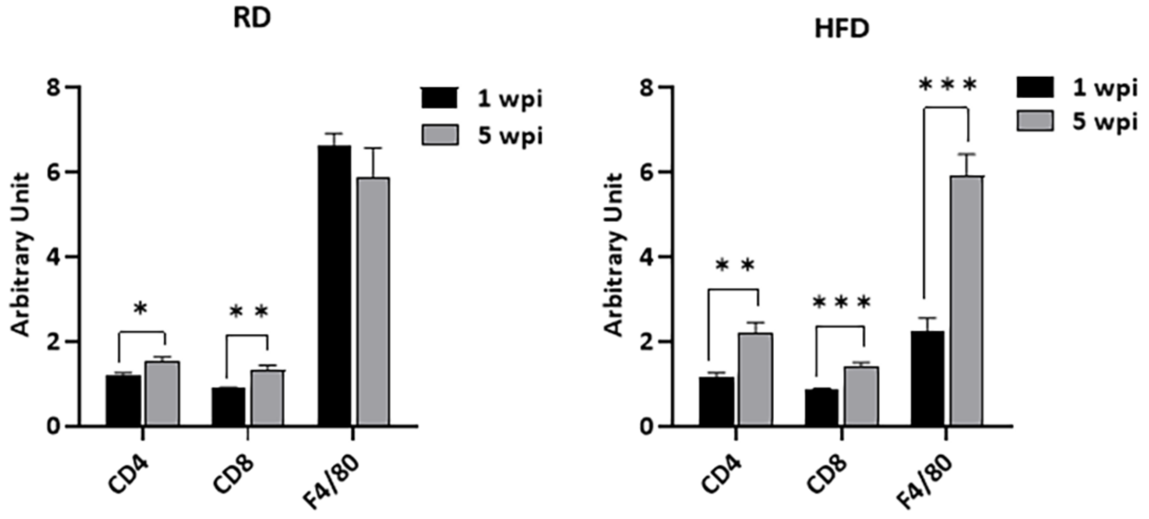

Figure S3: Bar graphs showing the levels of infiltrated immune cells (CD4, CD8 and F4/80 macrophages) normalized to β-Actin between 1 wpi and 5 wpi in both RD (left) and HFD (right) mice tumors. The error bars represent the standard error of the mean. * $p \leq 0.05$, ** $p < 0.005$ and *** $p \leq 0.001$.

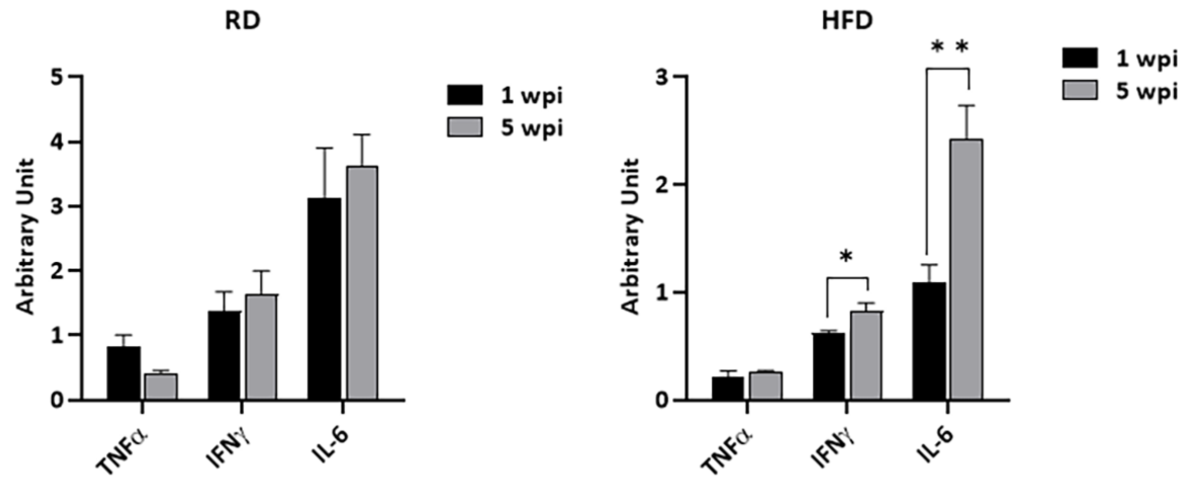

Figure S4: Bar graphs showing the levels of inflammatory markers (TNFα, IFNγ and IL-6) normalized to β-Actin between 1 wpi and 5 wpi in both RD (left) and HFD (right) mice tumors. The error bars represent the standard error of the mean. * $p \leq 0.05$ and ** $p \leq 0.005$.

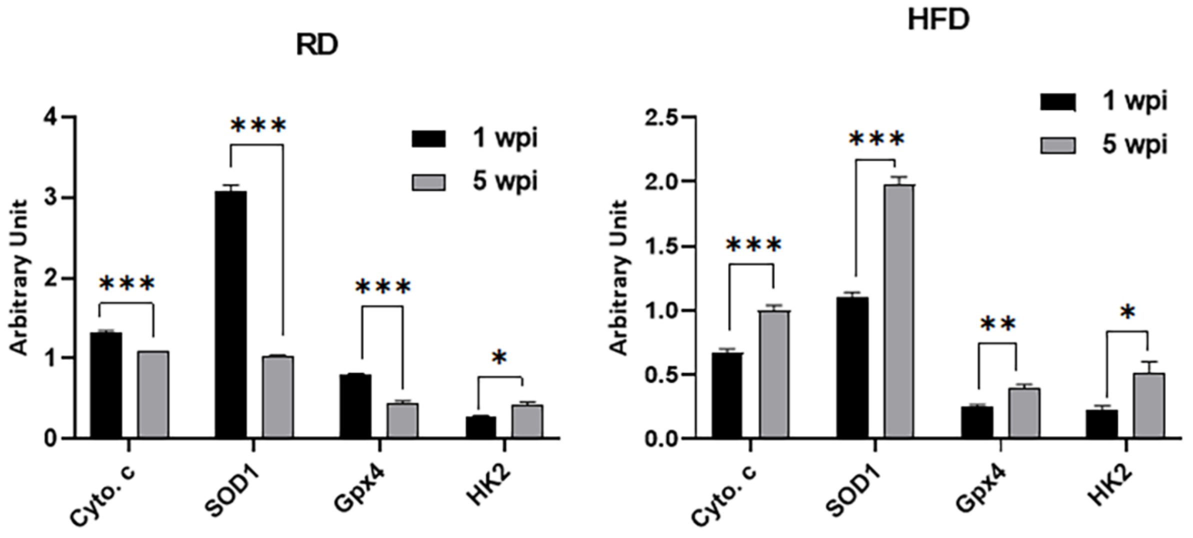

Figure S5: Bar graph showing the protein expression levels of energy metabolism (oxidative phosphorylation vs. glycolysis) normalized to β-Actin between 1 wpi and 5 wpi in both RD (left) and HFD (right) mice tumors. The error bars represent the standard error of the mean. * $p \leq 0.05$, ** $p \leq 0.005$ and *** $p \leq 0.001$.

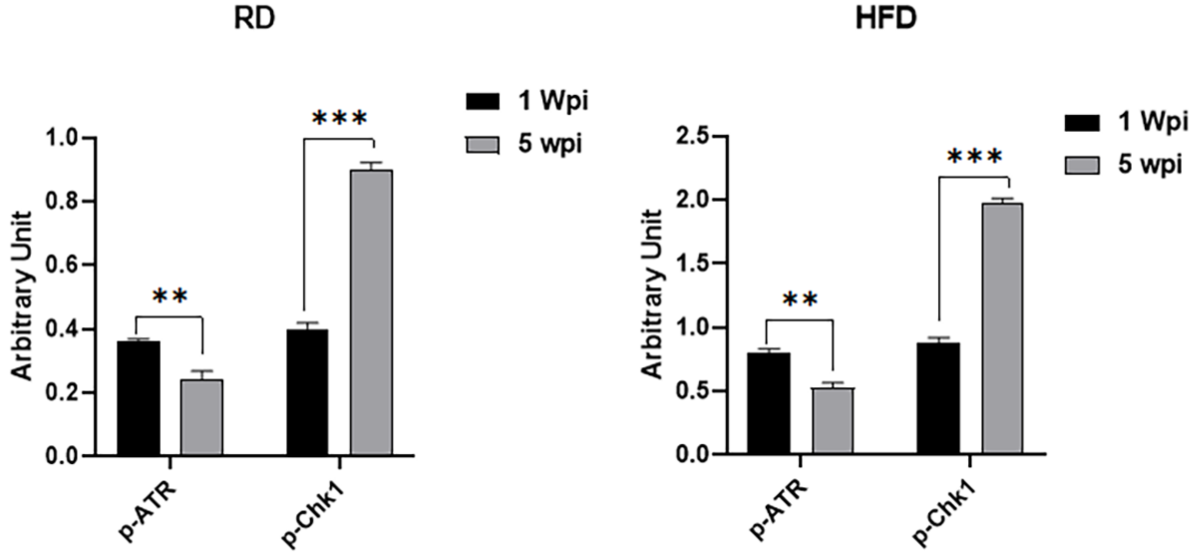

Figure S6: Bar graph showing the protein expression levels of DNA damage marker (p-Chk1 and p-ATR) normalized to β-Actin between 1 wpi and 5 wpi in both RD and HFD mice tumors. The error bars represent the standard error of the mean. ** $p \leq 0.005$ and *** $p \leq 0.001$.
